# A Brief Commentary on Functional Derangement of the Stomach in Indigestion

**Published:** 1842-07

**Authors:** 


Art. II.—*A Brief Commentary on Functional Derangement of the Stomach in Indigestion.* By William Wynter, m.d. Resident-Physician at Liverpool.—*Liverpool,* 1842. 24mo, pp. 25.

This is an incredible book: would to Heaven, as Dr. Johnson said of the piece of music, it had been an impossible one also! We have taken the trouble (God help us, poor critics!) actually to count the number of words in it, and they almost coincide with the number contained in four of the pages of this Journal! What *can* be the meaning of such a publication as this? The only one conceivable to us, with our present lights, is one so discreditable to any member of an honorable profession, that we will not adopt it without further scrutiny: it is, that this miserable atomy—shadow—imago—eidolon of a book has been printed merely to furnish a substratum to an advertisement in the newspapers, to announce to the world around the Mersey the portentous intelligence, that

Now is the winter of our discontent no longer to be found in "the county and borough of Brecknock," but is verily "Resident-Physician at Liverpool." But why *resident*-physician? Are the other physicians practising there, and practising so honorably and well, not resident also? Have our excellent friends, Drs. Jeffrey and Scott, and Macrorie and Baird become denizens of the steam-boats that crowd the Mersey, or of the steam-carriages that shoot along the Birmingham Railway?—But we have already wasted too much space on a production which is in every respect inconceivably paltry and despicable, and of which it is difficult to say whether the language is more inaccurate or the matter more absurd.

